# Perspectives on Fruit and Vegetable Consumption and Government Dietary Guidelines: Content Analysis of Comments on News Websites

**DOI:** 10.2196/19917

**Published:** 2021-08-19

**Authors:** B Hornsby, H Ensaff

**Affiliations:** 1 Nutritional Sciences and Epidemiology University of Leeds Leeds United Kingdom

**Keywords:** medical news, online news, user comments, public health, population health, qualitative analysis, perspectives, dietary guidelines, diet, fruit and vegetable consumption, mobile phone

## Abstract

**Background:**

News websites are an essential source of medical news for the public. Many websites offer users the opportunity to leave comments, which may provide rich insights into public perspectives on health issues. With an established role in public health, fruit and vegetable (FV) consumption is central to the government’s dietary guidelines. However, FV intake continues to fall short of government recommendations.

**Objective:**

Using comments from news websites, this study aims to explore public perspectives on FV intake and related government dietary guidelines.

**Methods:**

Data comprised 2696 web user comments generated in response to substantial media coverage for a meta-analysis examining FV consumption and the risk of all-cause mortality, cardiovascular disease, and total cancer. Using an inductive thematic approach, the data were analyzed and coded in an iterative process.

**Results:**

Four overarching themes emerged: personal factors, rejection, lack of knowledge, and food landscape, each with component subthemes. The lack of clarity around government dietary health guidelines was apparent, and this, along with emergent personal factors, may hinder better consumption. Rejection was also evident, as was a quality versus quantity of life debate.

**Conclusions:**

There are gaps in the public’s understanding of government guidelines, which may act as a constraint to better compliance. Further work should examine this issue and rejection and the possibility of public fatigue related to dietary health information and news. Similarly, future work should also explore targeted interventions with a specific emphasis on health literacy.

## Introduction

### Background

At present, mass media is popular and influential in society, with 95% of adults reportedly following the news [1]. There is also a growing acknowledgment of the media’s role in framing public health issues [2]. Web-based media platforms are an influential news source, and 60% of adults (16-34 years) follow the news through internet or mobile apps [1]. Therefore, this provides an opportunity to explore public perspectives related to public health issues. Specifically, web-based comments in response to media-reported medical research and news can act as a rich source of data for documenting public response. Previous studies have analyzed web-based news comments to explore perceptions related to weight loss surgery [3], dietary risks [4], human papillomavirus vaccination [5], and COVID-19 [6]. Similarly, a previous work [7] has also identified clear advantages with this approach, such as the immediacy of responses that reflect current attitudes (as opposed to recollected opinions). Furthermore, prior studies have reported the value of this approach in assessing public opinion [8] and revealing public perceptions [9].

The consumption of fruit and vegetables (FVs) has been established as a key element in public health dietary guidelines. Indeed, epidemiological evidence highlights FV intake in disease prevention [10-12] and supports its distinction within public health agendas. The UK dietary guidelines on FV consumption focus on *at least five portions of a variety of FVs each day*. This advice is based on the World Health Organization’s recommendation of a minimum of 400 g daily to reduce the risk of coronary heart disease, stroke, and some types of cancer [13]. Despite this, the National Diet and Nutrition Survey reveals inadequate intake, with only 31% of adults aged 19-64 years and 26% of adults aged ≥65 years meeting the *5 A Day* guidelines [14]. For children, the levels are worse, with only 8% of those aged 11-18 years meeting the guidelines, with a mean intake of 2.7 (SD 1.5) portions per day [14]. Low FV consumption is also an international issue [15], including among children [16-18].

Multiple models and theoretical frameworks relate to the public’s perspectives on FV intake, relevant guidelines, and food choice parameters. In particular, the socioecological model [19] emphasizes the interrelationship between individuals and the environment, as well as the many levels of influence on behavior. The most proximal level comprises an individual’s setting and their interactions with those nearest, such as family members, whereas more distal levels can capture interactions and settings, as well as influential social and cultural values and customs. The model captures how a range of aspects can influence an individual’s behavior, encompassing factors such as age, gender, income, and the home environment and external influences, such as food policy and retailers. The socioecological model has been used to consider children’s FV intake [20], children’s obesogenic dietary intake [21], barriers and enablers to healthy eating in young adults [22], FV consumption among low-income groups [23], and food choices of older adults [24]. Another useful theoretical consideration is the theory of planned behavior [25], which has been used previously to understand FV consumption [26-28]. The theoretical framework describes how attitude, subjective norm, and perceived behavioral control drive individual intention, and how intention strongly influences a particular behavior.

Previous research investigating FV determinants has pointed to taste preferences, time, availability, habit, motivation, and knowledge [29-31]. Evidence also points to general awareness for the *5 A Day* message but highlights the need for clearer details on, for example, portion sizes and variety [32]. Similarly, a prior study reported an association between low FV consumption and low knowledge of details pertaining to the *5 A Day* message [33].

### Objectives

With the general public’s increased consumption of web-based news, user comments can provide a unique data set to explore the public perspectives on FV intake. Indeed, a substantial number of web-based comments were generated following considerable press coverage related to a specific research publication [34]. Aune et al [34] conducted a meta-analysis of 95 studies and found that for all-cause mortality, coronary heart disease, stroke, and cardiovascular disease, the lowest risk was observed with an FV intake of 800 g/day (ie, twice the minimum *5 A Day* UK recommendation). Furthermore, the authors estimated that 7.8 million premature deaths globally in 2013 may be attributable to an intake of <800 g/day, if the associations observed are causal. The aim of this study is to examine web-based comments associated with media coverage (for this meta-analysis [34]) and to explore public perspectives on FV consumption and relevant dietary guidelines.

## Methods

### Data Collection and Analysis

The top 20 web-based media publications in the United Kingdom (by pageviews) were identified [35]. Foreign language and news aggregator sites were excluded, and the remaining sites were reviewed for web-based articles reporting the findings of previous research [34]. Relevant articles were checked for features that allowed web-based readers to post comments, and these were included in this study.

The news websites had comparable formatting, typified by the news article followed by a comments section, where readers could post their views and opinions. Standard practice dictates that users create an account with a unique username to post a comment. Thus, users can remain anonymous and protect their privacy on the internet. A relevant feature of web-based comments is the ability for commenters to *reply* to others, thereby creating conversations and generating discussions on a specific user-generated topic. Readers without an account, commonly referred to as *guests*, are still able to read others’ comments but are unable to post comments. Webpages have standard rules for comments to be published, and users could “report” comments, and those comments deemed offensive or inappropriate were removed by moderators.

All web-based comments for each identified media webpage were sourced, copied (as posted; ie, with spelling, grammar, and expletives unedited), and pasted into a separate document. Some posts were apparently removed by moderators; these were not included in this study. Once compiled, all comments were anonymized, that is, unique identifiers were substituted for usernames and pseudonyms for any identifying details. Anonymized comments were imported into NVivo 11 (QSR International) for data exploration and analysis. Web-based comments were analyzed using an inductive thematic approach. The analysis began with familiarization, where all comments were read and reread. An initial set of themes to capture user comments was created; themes were named appropriately and based on key insights emergent from the early analysis. Next, each source file was analyzed, and each comment was considered sequentially and coded to capture the essence of the comment. The coding was completed for all data to ensure that no comments were lost during the analysis, and all viewpoints were captured. Where data did not relate to any of the initial themes that had been identified during familiarization, then a new theme was considered and added. Thus, all the data were coded appropriately. After this initial round of coding, themes and their respective data were discussed among researchers. Themes were reviewed to ensure that they were a clear reflection of the data. Wherever necessary, themes were modified, for example, some themes merged into others and some secondary themes were created from primary themes. A second round of coding was completed. As a result, some comments were recoded into a newly introduced theme (which more accurately represented the essence of the comment). After this round of coding, themes were once again assessed, discussed, and reviewed before a third round of coding. After this iteration, it was felt that the endpoint of the coding had been reached and the final set of themes represented and described the data well. Researchers undertook several steps to maintain rigor in the analysis; these included scrutiny of the data coding and a detailed discussion of emergent themes. Researchers also practiced reflexivity [36], acknowledging their impact and preconceived perceptions and referencing these during researcher discussions.

### Ethical Considerations

Research using qualitative analysis of web-based comments to analyze public perspectives and perceptions is growing. Therefore, the requirements and protocols for this methodology are still being developed. This study was informed by practices in previous studies and ethical considerations for related internet-mediated research [37]. Although commenters would not have been aware that their comments would be used for research purposes, all comments collected in this study were posted on publicly available websites. Furthermore, all comments were anonymized and any identifying words were replaced before the data analysis.

## Results

### Overview

**Figure 1:**
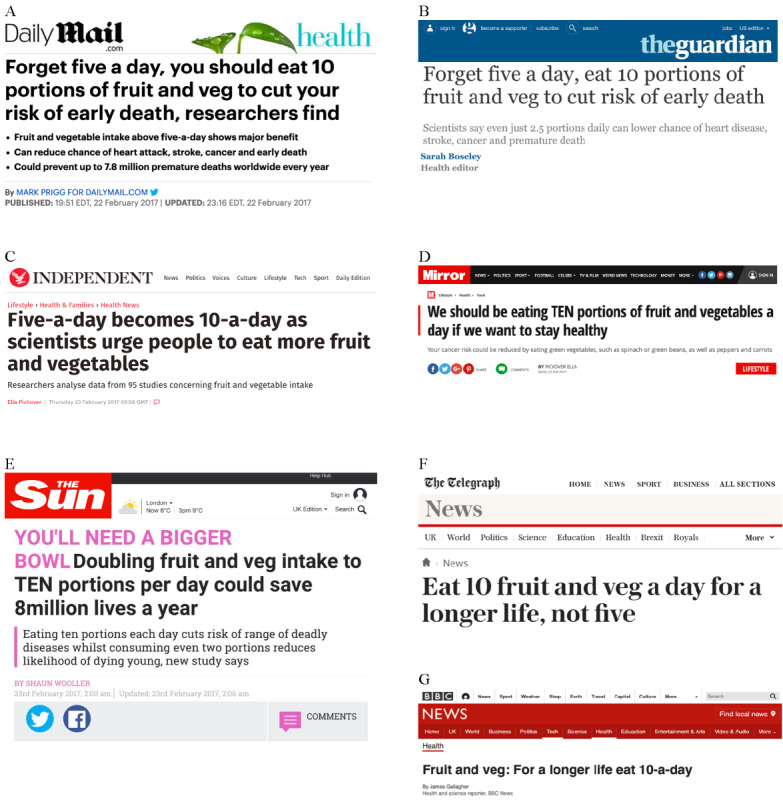
Selection of the mastheads and headlines from the news websites providing comments for this study: (A) Mail Online (image credit: Mail Online); (B) The Guardian (courtesy of Guardian News & Media Ltd); (C) Independent (image credit: ESI Media); (D) Mirror (image credit: Mirrorpix/Reach Licensing); (E) The Sun (image credit: The Sun/News Licensing); (F) The Telegraph (image credit: Telegraph Media Group); (G) BBC News (image credit: BBC News).

Overall, 2696 comments (103,930 words) were collected. Table 1 provides the details of the internet-based media publications (page views and readership demographics) and data (number of comments collected and source article headline). It is noticeable that all web-based news headlines referred to eating 10 portions a day. Figure 1 presents a selection of the mastheads and headlines.

Four main themes alongside their component subthemes emerged during data analysis (Textbox 1). Each theme and subtheme are considered alongside representative quotations.

**Table 1:** Details for the internet-based media publications and data included in this study (N=2696).

Web-based media	Domain pageviews (in millions), n^a^	Readership demographics^b^					Comments, n (%)		Article headline and reference
		Readers (in thousands), n	Male:female	≥35 years:15-34 years	ABC1:C2DE^c^				
BBC News	1715.5	—^d^	—	—	—	175 (6.49)		Fruit and veg: For a longer life eat 10-a-day [38]	
The Guardian	331.5	24,363	0.87	1.27	1.80	2369 (87.87)		Forget five a day, eat 10 portions of fruit and veg to cut risk of early death [39]	
Mail Online	318.7	26,937	0.79	1.34	1.59	83 (3.08)		Forget five a day, you should eat 10 portions of fruit and veg to cut your risk of early death, researchers find [40]	
The Telegraph	169.5	25,464	0.89	1.44	1.66	19 (0.70)		Eat 10 fruit and veg a day for a longer life, not five [41]	
Independent	93.9	22,755	0.80	1.41	1.65	17 (0.63)		Five-a-day becomes 10-a-day as scientists urge people to eat more fruit and vegetables [42]	
Express	61.3	12,530	1.20	1.64	1.72	1 (0.04)		5? No, you have to eat TEN portions of fruit and vegetables to keep healthy: Latest advice [43]	
Yahoo News	56.4	—	—	—	—	20 (0.74)		Now it’s TEN fruit and veg a day: Experts issue new guidance for staying healthy [44]	
Mirror	55.7	23,910	0.92	1.26	1.52	5 (0.19)		We should be eating TEN portions of fruit and vegetables a day if we want to stay healthy [45]	
The Sun	46.2	25,026	0.83	1.06	1.49	5 (0.19)		Doubling fruit and veg to TEN portions per day could save 8 million lives a year [46]	
Business Insider	36.7	—	—	—	—	2 (0.07)		Why you should eat 10 portions of fruits and vegetables a day instead of five [47]	

^a^Total pageviews for domains in February 2017 over desktop and mobile [35].

^b^Monthly audience estimates via a PC or via a browser or an app on a device (smartphone or tablet) July 2016-June 2017 [48].

^c^Classification system based on the occupation of the household’s chief income earner (ABC1 corresponds to a combination of the more advantaged socio-economic groups, and C2DE corresponds to a combination of the less advantaged socio-economic groups).

^d^Information missing for BBC News, Yahoo News, and Business Insider.

**Emergent themes and subthemes from analysis of comments on news websites.**

**Personal Factors**

- Children
- Taste
- Time, effort, and skill

**Rejection**

- Ridicule
- Skepticism
- Quality versus quantity of life

**Lack of Knowledge**

- What counts as fruits and vegetables (FVs), and how much is a portion?
- Limited health knowledge

**Food Landscape**

- FV cost
- FV availability

### Personal Factors

#### Children

Commenters discussed various influences on FV intake, and within these, several factors emerged: children; taste; and time, effort, and skill. Often, these factors were discussed as barriers, for example, how to overcome difficulties and “to get children to eat much fruit and veg”:

My kids would tell you they hate mushrooms and don’t eat them but they actually eat them all the time without knowing it because I chop them really finely in things like pasta sauce!

The importance of health education, healthy diets, and FV consumption during childhood was also highlighted:

I believe a lot of the problems begins when you’re a child. What you pick up from your parents then, determines how you behave later on.

If you can’t manage it yourself, make sure you feed it to your children from an early age and be a bit tough with them about it. Once they’ve got through their pernickety hurdles, they’ll be healthy veg eaters all their lives.

#### Taste

The importance of taste was discussed, with some referring to unpalatable flavors:

If vegetables are so good for you then why do they taste so horrible?

make fruit and veg more tasty and you got yourself a deal

In contrast, other commenters spoke positively and recounted food practices:

There are few things quite as delicious as a stir fry or a stew with maybe 5 or 6 different veggies in. Trust me, tastes good!

#### Time, Effort, and Skill

Commenters also referred to other constraints, including the time and effort required, as well as personal cooking skills:

Eating 10 a day won’t actually increase your lifespan but it will certainly feel like it with the time taken to source, prepare, eat it...

Where the hell do you expect a low-income mum who herself grew up eating frozen lasagna ready meals to summon the wherewithal to buy quite costly fresh ingredients and have time and skill to cook them into tasty meals?

I do acknowledge that if you either cannot cook or are very strapped for time, it’s much harder to do [eat 10 a day] with shop-bought ready food only. Yet another reason why food education and learning to cook is so important.

### Rejection

#### Ridicule

A key theme of this study was rejection. This included ridicule, which was mainly referred to through comments relating FV to alcohol and fast food:

Does my glass of wine a night count as 1 a day?

Grapes are fruit, so drink wine. Hops and barley are vegetables, so drink beer. Cocoa is a plant, so eat chocolate. Cows are herbivores, so eat burgers. Sorted.

Too time consuming; a Big Mac followed by a Wagon Wheel and washed down with a can of something is quicker.

#### Skepticism

Skepticism emerged as a second subtheme within rejection, relating to the reporting, the research itself, and dietary guidelines:

No doubt the news tomorrow will be that eating 10 a day gives you cancer.

Immediately the media turn this reported research into a requirement to eat 10 portions a day. No wonder everyone is confused or scared by the way health matters are reported.

Sorry but this study is nonsense. They asked 65,000 people what fruit and veg they ate in the previous 24h, nothing else about any other time. They then looked at the 4400 who died in the next 7-8 years and predicted the results based on 24 h of their eating. How can that then be used to predict outcomes over a lifetime of eating?

First it was eat some fruit & veg every day to live longer, then eat 5x fruit & veg to live longer, now it’s eat 10x fruit & veg to live longer, I’m going to just continuously eat fruit and veg (via drip whilst sleeping), I recon I’ll be good for the first person to 200!! Unless I get diabetes and need to fast anyway...I wonder what we’re going to be told to eat/do tomorrow?

5-a-day - plucked out of the air. 10-a-day - plucked out of the air. 28 units of alcohol a week - plucked out of the air. 2 litres of water a day - plucked out of the air. 10,000 steps a day - plucked out of the air. Government health advice - all plucked out of the air.

#### Quality Versus Quantity of Life

Interestingly, a debate between the quality and quantity of life emerged:

I’m fed up with people harping-on about longevity. Surely the aim of a healthy diet and lifestyle is to improve the overall quality of life, not merely its length.

Healthy eating...great I’m all for it. But why the obsession about living longer? I’d rather die at 80 with some dignity, sanity, and independence than live to be 110 as a pale shadow of my former self.

### Lack of Knowledge

#### What Counts as Fruits and Vegetables (FVs), and How Much is a Portion?

This theme encompassed uncertainty related to FV portion sizes and whether certain foods are counted as FV in the dietary guidelines:

They really, really, really need to stop using the word “portions” and “servings”. They are vague and mean little to most punters. How many apricots or carrots are there in a portion? How many portions in a bowl of salad? Clearer language is needed in communicating this stuff.

I do already eat a lot of mixed nuts. I’m unsure as to whether they count as vegetables, though.

#### Limited Health Knowledge

The analysis also revealed a gap in diet and health knowledge more generally:

5 or more oranges a day is a huge amount of instantly available fructose and as bad as eating 2 Mars bars daily

Mmmm, pesticides and GMOs. Cancerlicious!

Ten a day and you will die of pesticides no doubt

### Food Landscape

#### FV Cost

The wider food landscape emerged as a theme with respect to FV. The cost of FV was highlighted, with comments referring to FV as expensive, citing everyday monetary realities:

All well and good but extremely divorced from the reality of everyday life and all its struggles as many cannot afford more then a couple of portions of fruit and veg a day at most

...the price of fresh fruit has gone through the roof, the supermarkets are getting beyond a joke now with their pricing. With the govt cutting benefits for everyone who needs them makes veg and fruit unaffordable.

In contrast, some comments suggest that prudent shopping habits are an effective way to counter the barrier of cost:

Go to the frozen section in more or less any supermarket, you can get a 1 kg bag of mixed veg for about 80p.

Get down the supermarket and find out the times they yellow label stuff. Just ask, most of them do it at fairly regular times. You can get tons of fruit and veg on the cheap just by lurking at the right times

#### FV Availability

The second subtheme within the overarching food landscape is FV availability. This subtheme related to the available quality and variety, for example, *"limited choice, very poor quality"*, and also *highlighted seasonality*:

...fruit and vegetables which only kept fresh a couple of days. I Often have to throw away soft fruit with mould after a couple of days.

How can we eat 10 portions of seasonal veg without getting bored of turnips and swede?

## Discussion

### Principal Findings

The analysis revealed an array of factors relating to public perspectives on FV and reflected the complex nature of food choices and dietary habits. Specifically, FV consumption was affected by the wider food landscape. This resonates with the more distal levels of influence in the socioecological model. Commenters reported that low FV intake was influenced by the produce available in retailers; these comments referred to poor quality, limited variety, and poor shelf life. Similarly, cost was highlighted and there was a general perception that FV is a high-price commodity, with supermarket pricing “beyond a joke.” Monetary realities of daily life making FV unaffordable has been reported in previous research in low-income populations, which found that financial considerations impacted FV purchases of parents [49]. Similarly, comments relating to shopping budgets and wages to personal diets reflect the association between income and FV consumption [50], and cost as a barrier to FV intake [27,51,52] as well as associations between food insecurity and FV consumption [53].

A key finding of this study is the personal factors that commenters associated with FV consumption. This included encouraging children to eat FV; notably, parents referred to strategies to increase children’s FV intake. Other research has revealed parenting practices and FV availability [54], parents’ coping strategies such as playing games with food to make it more appealing [49], and children’s growing authority over everyday food [55].

Web-based commenters acknowledged the importance of parents’ roles and childhood food experiences. This resonates with individuals’ food histories and how previous food encounters (among other factors) contribute to current food choices, as depicted in the food choice process model [56,57]. Parents’ recognition of the importance, as shown in this study, is noteworthy. Further research to understand parents’ perspectives as food gatekeepers (with chief responsibility for food provision) and, in particular, their strategies and receptiveness to adopting new approaches is warranted. This would add to the valuable research in this area on effective and ineffective parental behaviors [58].

The emergence of time as a potential barrier implies the everyday pressures experienced by many, and the limited and declining time spent on meal preparation [59]. Similarly, this study highlights the effort and skill required. Furthermore, commenters attested to parents’ role in imparting cooking skills. Previous research has noted the relevance of food skills in promoting healthier food choices [60] and the relevance of children’s food skills [61,62].

One of the key findings of this study is the theme of rejection. This included ridiculing scientific research as well as references to alcohol and fast food. For some commenters, this may reflect an unwillingness to change dietary practices regardless of government health advice; however, further work is required to understand this observation. A noteworthy finding was the debate between quality of life and quantity. Indeed, this implies the potential of healthy eating messages from the perspective of enhancing the quality of life. Further consideration is warranted to establish whether this may be a useful public health strategy to improve dietary intake.

In this study, there was a sense of public messages being unclear and deterring efforts to change dietary habits. An emergent theme was the lack of knowledge, where many commenters expressed confusion related to the current *5 A Day* dietary guidelines. In particular, the portion sizes and what FV qualified were highlighted. This result relays a lack of clarity and a gap in understanding. This also aligns with other work [32,63] reporting confusion related to health messages.

A level of skepticism toward the media was observed from some comments, particularly the reporting of scientific research and health advice. The damage to public trust in nutrition from unrepresentative news stories [64] should not be underestimated. This may contribute to the public fatigue regarding diet and health agendas and underlines the importance of accurate and representative reporting. This is particularly the case, given the pivotal role that mass media play in relaying scientific research.

This study provides valuable insights and contributes to the growing literature using this methodology [3-9,65,66]. The web-based comments provided a rich data source for analyzing public perspectives and perceptions of FV. It is acknowledged that the originators of the data, web-based commenters, are likely to differ from the general population. They have a certain level of web literacy and consume web-based news. Indeed, by definition, they accessed web-based news articles and commented. Interestingly, a cross-national analysis found that 8% of web-based news users in the United Kingdom reported commenting on news websites during an average week [67].

Demographic details of the readership of web-based news media were available. However, the specific details of web-based commenters were not available. Therefore, their demographic characteristics could not be assessed, and this deficit has been acknowledged in similar studies [7,8]. Nevertheless, there is a distinct value in examining these types of data, not least because they do relay the perceptions and perspectives of a portion of the population. Furthermore, some of the value of examining web-based comments can be attributed to the unique features of the data. For example, the anonymity afforded by web-based comments (compared with a research interview) may foster less-filtered data. Indeed, a US study [68] reported that two-third of web-based news consumers felt that anonymity allowed commenters to express ideas that they might otherwise fear expressing. Therefore, although the specific characteristics of web-based commenters are hidden, capturing such perceptions is worthwhile. Web-based comments also have the advantage of being direct responses instigated by users (as opposed to being requested by a researcher). They are within the users’ own domain, independent of researchers and without their influence. Similarly, a discussion between commenters may be less constrained, and a relevant feature was commenters *replying* to others’ comments, creating a dialog that was beneficial for gathering a range of viewpoints surrounding a topic, for example, FV cost.

### Limitations

It is important to acknowledge this study’s limitations. Comments were sourced from 10 media outlets. However, most data originated from a single source; therefore, the findings may be specific to this readership. Demographic information for the news media provides some contextual information and may reflect, to some degree, the web-based commenters; however, there is a large degree of uncertainty surrounding the identity and characteristics of the commenters. Some commenters may portray themselves differently on the internet than in *real life*. For example, previous research has indicated that internet bloggers take on multiple personas across multiple types of social media [69]. Furthermore, given the global reach of web-based news media, all commenters may not have been based in the United Kingdom; indeed, there was evidence of some comments originating from further afield. Automated news commentary [70] is acknowledged, and although all comments in this study were reviewed manually, comment authenticity may have been compromised by bot comments.

### Implications for Research and Practice

This study provides valuable insights regarding the public perspectives on FV and related dietary guidelines. Further research combining this approach with more traditional approaches would be beneficial. Similarly, incorporating data from multiple social media platforms is recommended to provide a greater understanding of relevant issues.

This study has implications for health policies and practices, as well as future interventions. Bearing in mind the limitations outlined earlier, findings more broadly point to the need to address the public’s health literacy. To some extent, this may also address other barriers, such as time and FV costs. The findings also highlight the need for a better understanding of government guidelines and the need for improved skills. Optimistically, commenters’ perceptions of the relevance of food skills were revealed. Incorporating food skills within interventions warrants further investigation. Previous work [71] has identified education through cooking classes can be beneficial for increasing FV consumption. This also resonates with the theory of planned behavior and, specifically, harnesses perceived behavioral control to drive individual intention to influence behavior.

This study highlights the cost and availability of FVs. Given the suboptimal FV consumption (and its place within population health), addressing these factors is vital. Future studies should explore schemes offering discounts to encourage higher consumption. Previous work has indicated that supermarket discount interventions can increase consumer FV purchases and consumption [72]. Furthermore, health interventions may instigate individual-led changes in the home environment. This is commensurate with reciprocal determinism, a concept relevant to the socioecological model, whereby behavior and environment influence each other and individuals can themselves influence the environment.

Similarly, the relevance of parents, as highlighted in this study, needs further consideration. This is particularly relevant for targeted interventions. Finally, the findings related to rejection and skepticism point to the important role that news websites play in informing the public about medical research and news. Furthermore, they underline the importance of representative and accurate reporting of health issues.

### Conclusions

A range of factors are relevant in understanding the public’s perceptions and perspectives on FV intake. Some relate to the external food environment, whereas others relate to food skills and health knowledge. There is a need to examine the nation’s health literacy and its potential role in supporting positive dietary change (specifically FV intake).

## Abbreviations

FV: fruit and vegetable
